# Nontypeable *Haemophilus influenzae *induces COX-2 and PGE2 expression in lung epithelial cells via activation of p38 MAPK and NF-kappa B

**DOI:** 10.1186/1465-9921-9-16

**Published:** 2008-01-31

**Authors:** Feng Xu, Zhihao Xu, Rong Zhang, Zuqun Wu, Jae-Hyang Lim, Tomoaki Koga, Jian-Dong Li, Huahao Shen

**Affiliations:** 1 Department of Respiratory Medicine, Second Affiliated Hospital, Zhejiang University School of Medicine, Hangzhou, Zhejiang 310009, China; 2 Center of Clinical Laboratories, Second Affiliated Hospital, Zhejiang University School of Medicine, Hangzhou, Zhejiang 310009, China; 3 Department of Microbiology & Immunology, University of Rochester Medical Center, Rochester, 14642, USA

## Abstract

**Background:**

Nontypeable *Haemophilus influenzae *(NTHi) is an important respiratory pathogen implicated as an infectious trigger in chronic obstructive pulmonary disease, but its molecular interaction with human lung epithelial cells remains unclear. Herein, we tested that the hypothesis that NTHi induces the expression of cyclooxygenase (COX)-2 and prostaglandin E2 (PGE2) via activation of p38 mitogen-activated protein kinase (MAPK) and nuclear factor (NF)-kappa B in pulmonary alveolar epithelial cells.

**Methods:**

Human alveolar epithelial A549 cells were infected with different concentrations of NTHi. The phosphorylation of p38 MAPK was detected by Western blot analysis, the DNA binding activity of NF-kappa B was assessed by electrophoretic mobility shift assay (EMSA), and the expressions of COX-1 and 2 mRNA and PGE2 protein were measured by reverse transcription-polymerase chain reaction (RT-PCR) and enzyme linked immunosorbent assay (ELISA), respectively. The roles of Toll-like receptor (TLR) 2 and TLR4, well known NTHi recognizing receptor in lung epithelial cell and gram-negative bacteria receptor, respectively, on the NTHi-induced COX-2 expression were investigated in the HEK293 cells overexpressing TLR2 and TLR4 *in vitro *and in the mouse model of NTHi-induced pneumonia by using TLR2 and TLR4 knock-out mice *in vivo*. In addition, the role of p38 MAPK and NF-kappa B on the NTHi-induced COX-2 and PGE2 expression was investigated by using their specific chemical inhibitors.

**Results:**

NTHi induced COX-2 mRNA expression in a dose-dependent manner, but not COX-1 mRNA expression in A549 cells. The enhanced expression of PGE2 by NTHi infection was significantly decreased by pre-treatment of COX-2 specific inhibitor, but not by COX-1 inhibitor. NTHi induced COX-2 expression was mediated by TLR2 in the epithelial cell *in vitro *and in the lungs of mice *in vivo*. NTHi induced phosphorylation of p38 MAPK and up-regulated DNA binding activity of NF-kappa B. Moreover, the expressions of COX-2 and PGE2 were significantly inhibited by specific inhibitors of p38 MAPK and NF-kappa B. However, NTHi-induced DNA binding activity of NF-kappa B was not affected by the inhibition of p38 MAPK.

**Conclusion:**

NTHi induces COX-2 and PGE2 expression in a p38 MAPK and NF-kappa B-dependent manner through TLR2 in lung epithelial cells *in vitro *and lung tissues *in vivo*. The full understanding of the role of endogenous anti-inflammatory PGE2 and its regulation will bring new insight to the resolution of inflammation in pulmonary bacterial infections.

## Background

Nontypeable *Haemophilus influenzae *(NTHi) is one of common and important respiratory pathogens. NTHi causes otitis media and conductive hearing loss in children while pulmonary presence of this facultative intracellular pathogen is implicated as an infectious trigger in chronic obstructive pulmonary disease (COPD) in adults [1,2]. The emergence of antibiotic-resistance strains of NTHi and the difficulty of development of efficacious vaccines urge further efforts to understand the host response mechanisms involved in NTHi infections.

The respiratory epithelium is an important interface to environmental microorganisms. In addition to provide a physical barrier against microbial invasion and contribute to mucociliary clearance, respiratory epithelial cells are actively involved in inflammation and host defense of the lung in multiple ways. In particular, type 2 alveolar epithelial cells (AECs) as a defender of the alveolus are located in alveoli where they recognize invading pathogens by extracellular and intracellular receptors and contribute to host innate immunity [3-5]. Lipid metabolites of arachidonic acid such as prostaglandins have been shown to modulate immune and inflammatory responses [6,7]. Prostaglandin E2 (PGE2) is a product of the cyclooxygenase (COX) pathway. Two isoforms of COX, the constitutively expressed COX-1 and the inducible COX-2, have been identified. PGE2 is commonly thought to have proinflammatory effects on the pathogenesis of several inflammatory diseases including rheumatoid arthritis and periodontitis [7,8]. However, increasing evidence demonstrated that pulmonary PGE2 has a role in limiting the inflammatory response and tissue repair in contrast to its counterparts in other organs of the body [7].

The expression of COX-derived PGE2 and its molecular regulation depend on cell types and stimuli [9]. In the present study, we showed that NTHi induced COX-2 expression and subsequent PGE2 production via activation of p38 mitogen-activated protein kinase (MAPK) and nuclear factor (NF)-kappa B in lung epithelial cells. The full understanding of the role of pulmonary endogenous anti-inflammatory mediators such as PGE2 and their regulation will bring new insight and develop novel treatment aiming at immune modulation.

## Methods

### Materials

SB203580, SB202190, PDTC, SC560, and NS398 were purchased from Sigma Chemicals (CA, USA), PGE2 ELISA kit was from R&D Co. (Minneapolis, USA). All other chemicals used were of analytical grade and obtained from commercial sources.

### Isolation and identification of bacterial strain

NTHi strain was a clinical isolate from Second Affiliated Hospital of Medical School, Zhejiang University. The suspectable *H. influenzae *strains were confirmed by X, V and X+V factor requirement test, satellite test and API-NH identification system. Slide serum agglutination test was performed and the isolated strain was proved to not agglutinate with all the capsule antiserum of type a, b, c, d, e, and f. Finally, the isolated strain was identified by 16S rRNA gene amplification and sequencing. NTHi strain 12 was used for *in vitro *HEK239 cell experiments and *in vivo *mice experiments.

### Mice experiments

C57BL/6 and BALB/c mouse strains, background strain for TLR2 and TLR4 knock-out, respectively, and TLR2 and TLR4 knock-out mice were used for NTHi-induced COX-2 expression in NTHi-induced pneumonia model in mice *in vivo*. C57BL/6 mice were purchased from National Cancer Institute (NCI, NIH, USA) and TLR2 knock-out mice were kindly provide by Dr. S. Akira, and TLR4 knock-out mice were purchased from Jackson Lab. (USA). Under the anesthesia, wild-type and TLR2 and TLR4 knock-out mice were intratracheally inoculated with 1 × 10^7 ^CFU of NTHi strain 12. Mice lung tissues were collected 6 h after NTHi inoculation and mRNA expression of COX-2 was measured by quantitative RT-PCR (Q-PCR) as described below. All the animal experiments were approved by the Institutional Animal Care and Use Committee at University of Rochester.

### Cell culture and in vitro experiments

A human alveolar epithelial cell line A549 (ATCC-CCL-185) was a kind gift from Shanghai ZJ Bio-Tech Co. Ltd., China. A549 cells were grown in 75 cm^2 ^polystyrene flasks with RPMI1640 (HyClone, Tauranga, New Zealand) supplemented with 10% heat-inactivated fetal calf serum (Gibco, NY, USA). A549 cells were seeded at 1 × 10^6 ^cells per well of 6-well flat-bottom, cell culture plates (Corning, NY, USA). This produced a confluent monolayer after overnight incubation at 37°C in a 5% CO_2 _humidified atmosphere. After growth medium was replaced by an antibiotic-free medium, A549 cells (1 × 10^6 ^cells/mL) were infected with NTHi (multiplicity of infection, MOI: 1, 10, 50). Inhibition experiments were carried out by 1 h pretreatment with the p38 MAPK inhibitor SB203580 (20 μM), SB202190 (10 μM), the NF-kappa B inhibitor PDTC (40 μM) or the selective COX-1 inhibitor SC560 (5 μM), COX-2 inhibitor NS398 (10 μM) before bacterial stimulation. Supernatants after incubation were collected and stored at -70°C freezer for ELISA detection. HEK293 cells, stably overexpressing pcDNA, TLR2, and TLR4, were kindly provided by Dr. D.T. Golenbock, and cells were maintained in Dulbecco's modified Eagle's medium supplemented with 10% FBS, 0.5 mg/mL of G418, and 10 μg/mL of ciprofloxacin (Cellgro, Herndon, VA).

### Reverse transcription-polymerase chain reaction (RT-PCR)

Total RNA was isolated from A549 cells with Trizol Reagent (Invitrogen, CA, USA). For cDNA synthesis, 20 μL RT mixture containing total RNA 2 μg, dNTP 1 mM/L, Olig(dt)_17 _prime 0.2 μg, RNasin 20 U, M-MLV reverse transcriptase 200 U was incubated at 42°C for 60 min, then the reverse transcriptase was inactivated at 72°C for 15 min. PCR amplification was performed on a PE2400 cycler (Perkin-Elmer, Massachusetts, USA). Omiga 2.0 software (Oxford Molecular, Oxford, UK) was employed to design oligonucleotide primers specific for human COX-1, COX-2 and GAPDH (an internal control). COX-1: forward: 5' AGTACCGCAAGAGGTTTGGC 3', reverse: 5' GCCGTCTTGACAATGTTAAAGC 3'; COX-2: forward: 5' GACAGTCCA CCAACTTACAAT 3', reverse: 5' CATCTCTCCATCAATTATCTGAT 3'; GAPDH: forward: 5' GTCGGTGTGAACGGATTT 3', reverse: 5' ACTCCACGACGTACTCAGC 3', with the product sizes 292 bp, 411 bp and 276 bp, respectively. The condition of PCR reactions was used as follows: 94°C for 5 min, then 94°C for 1 min; 63°C for COX-1, 56°C for COX-2 and 57°C for GAPDH for 1 min; 72°C for 45 s for 30 cycles (GAPDH) or 40 cycles (COX-1, 2) and 72°C for 10 min to end the reaction. PCR products were electrophoresed by 1.5% agarose gel containing ethidium bromide.

### Quantitative reverse transcription-polymerase chain reaction (Q-PCR)

Total RNA was isolated by using Trizol Reagent (Invitrogen), and the reverse transcription reaction was conducted with TaqMan reverse transcription reagents (Applied Biosystems). PCR amplications were performed by using SYBR Green universal master mix for human and mouse COX-2. Reactions were amplified and quantified by using as ABI 7500 sequence detector. Relative quantity of mRNAs were obtained by using the comparative Ct method and was normalized by using TaqMan predeveloped assay reagent human cyclophilin and mouse GAPDH for human COX-2 and mouse COX-2, respectively. The primers for human COX-2 were as follows: forward: 5'GAATCATTCACCAGGCAAATTG 3' and reverse: 5' TCTGTA CTGCGGGTGGAA CA 3'. The primers for mouse COX-2 were as follows: forward: 5' CCAGCACTTCAC CCATCAGTT 3' and reverse: 5' ACCCAGGTCCTCGCTTATGA 3'.

### Western blot

A549 were harvested by scrapers after stimulation of 15 min and 30 min. The cell pellets were lysed in lysis buffer (125 mM Tris, pH 6.8, 4% SDS, 20% glycerol, 100 mM dithiothreitol, and 0.5% bromophenol blue) and heated for 5 min at 95°C. Electrophoresis was performed at 200 V for 1 h with 12% SDS-PAGE at room temperature. Proteins were transferred to nitrocellulose membrane at 75 V for 1.5 h by wet blot at 4°C in Mini-Protein (Bio-Rad, California, USA). The membrane was then blocked with 5% non-fat dried milk in T-TBS for 1 h, washed three times with T-TBS and incubated with phospho-p38 MAPK antibody (Cell Signaling, Bevery, USA) at 4°C overnight. The blots were washed three times with T-TBS and incubated for 1 h with HRP-conjugated goat-anti-rabbit IgG (Cell Signaling) at room temperature. Immunoreactive bands were developed using an ECL chemiluminescent substrate (Pierce, Rockford, USA). Autoradiography was performed with optimized exposure times. Accordingly, p38 MAPK (Cell Signaling) was detected simultaneously to confirm equal protein load.

### Electrophoretic mobility shift assay (EMSA)

After stimulation of A549 cells, nuclear protein was isolated using NE-PER Nuclear and Cytoplasmic Extraction Reagents (Pierce). Biotin-labeled consensus NF-kappa B oligonucleotides were purchased from Invitrogen (Shanghai, China). The sequence of oligo was: 5' AGTTGAGGGGACTTTCCCAGGC 3'. Briefly, EMSA binding reactions were performed by incubating 5 μg of nuclear extract with the annealed oligos according to the manufacturer's instructions (Lightshift EMSA Kit, Pierce). The reaction mixture was subjected to electrophoresis on a 5% native gel.

### Enzyme linked immunosorbent assay (ELISA)

PGE2 level in cell supernatants was measured using commercially available kits (R&D Systems) according to the manufacturer's protocol. The limit of PGE_2 _assay was 39–5000 pg/ml.

### Statistical analysis

All data were presented as means ± SEM. One-way ANOVA was used for statistical analysis of the differences between the groups. A value of *P *< 0.05 was considered statistically significant.

## Results

### NTHi-induced COX-2 expression in A549 cells

**Figure 1:**
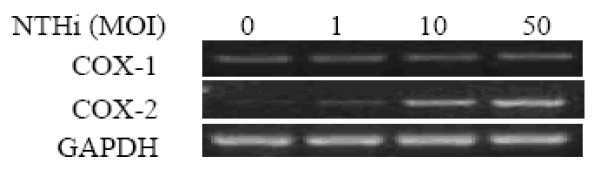
**The mRNA expression of COX-2 but not COX-1 induced by NTHi in a dose-dependent manner**. A549 cells were inoculated with different concentrations of NTHi, as indicated in the figure, and mRNA expression of COX-1 and COX-2 was measured by RT-PCR analysis.

A549 cells were inoculated with NTHi at a MOI between 1 and 50 for 4 h, and RT-PCR analysis performed for the mRNA expression. As shown in Fig. 1, NTHi inoculation dose-dependently induced expression of COX-2 mRNA, whereas had no effect on COX-1 mRNA in A549 cells.

### PGE2 release from NTHi-stimulated A549 cells in a COX-2 dependent way

**Figure 2:**
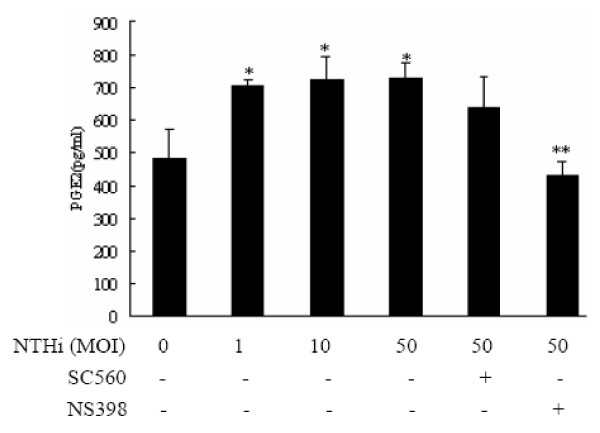
**NTHi-induced PGE2 expression mediated by COX-2 up-regulation**. A549 cells were inoculated with different concentrations of NTHi with or without SC560 (5 μM) and NS398 (10 μM), specific inhibitors of COX-1 and COX-2, and expression levels of PGE2 were measured by ELISA assay. Data are means ± SEM (n = 4), *p < 0.05 vs. Mock group; **p < 0.05 vs. NTHi group at 50 MOI.

To investigate the role of NTHi-induced COX-2 expression on the PGE2 production, A549 cells, first, were inoculated with NTHi at a MOI between 1 and 50 for 16 h, and PGE2 expression was measured using ELISA assay. Result demonstrated that PGE2 release from A549 cells was significantly increased by NTHi (Fig. 2). Furthermore, the pre-treatment of selective COX-2 inhibitor NS398 significantly decreased NTHi-induced PGE_2 _production to baseline levels, but pre-treatment of selective COX-1 inhibitor SC560 has no effect on NTHi-induced PGE2 release in lung epithelial cells A549. Taken together, these data indicate that NTHi-induced PGE2 production was mediated by COX-2 dependent pathway, but independently from COX-1 pathway.

### NTHi-induced COX-2 expression mediated by TLR2 signaling pathway

**Figure 3:**
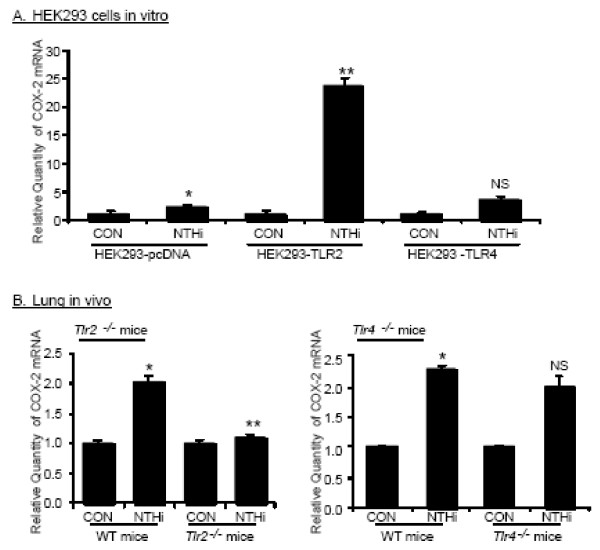
**COX-2 up-regulation mediated by TLR2 in epithelial cells *in vitro *and lung tissues of mice *in vivo***. **A**. HEK293 cells overexpressing pcDNA, TLR2, and TLR4 were inoculated with NTHi, and COX-2 mRNA expression was measured by Q-PCR analysis. Data are means ± SEM (n = 3). *p < 0.05 vs. CON; **p < 0.005 vs. NTHi in HEK293-pcDNA. NS:non-significant *vs*. NTHi in HEK293-pcDNA; CON: control. **B: **Wild-type and TLR2 and TLR4 knock-out mice were intratracheally inoculated with 1 × 10^7 ^CFU of NTHi, and mRNA expression of COX-2 was measured from the lungs of inoculated mice 6 h after inoculation. Data are means ± SEM (n = 3). *p < 0.05 *vs*. CON in wild-type mice; **p < 0.05 *vs*. NTHi in wild-type mice. NS: non-significant *vs*. NTHi in wild-type mice; CON: control.

Given the fact that TLR2 and TLR4 are of particular important in NTHi-induced signaling pathway and gram-negative bacteria-initiated signaling pathway in lung epithelial cells, respectively, we investigated the possible involvement of TLR2 and TLR4 in NTHi-induced COX-2 expression *in vitro *by using HEK293 cells overexpressing TLR2 or TLR4. As shown in Fig. 3A, NTHi-induced COX-2 expression was significantly enhanced by overexpressing TLR2, but not by overexpressing TLR4. To further investigate the role of TLR2 and TLR4 in NTHi-induced COX-2 expression *in vivo*, wild type, and TLR2 and TLR4 knock-out mice were intratracheally inoculated with 1 × 10^7 ^CFU of NTHi strain 12, and mRNA expression of COX-2 in lungs of infected mice were measured by Q-PCR analysis 6 h after inoculation. As shown in Fig. 3B, NTHi induced COX-2 expression in lung tissues of wild-type mice, and NTHi-induced COX-2 expression was abolished by TLR2-deficiency in TLR2 knock-out mice, but not by TLR4-deficiency in TLR4 knock-out mice. Taken together, these data indicate that NTHi-induced COX-2 expression is mediated by TLR2-dependent pathway, but independently from TLR4.

### p38 MAPK instantly activated by NTHi

**Figure 4:**
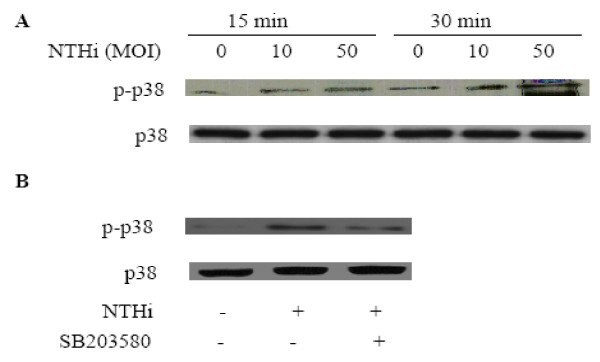
**Phosphorylation of p38 MPAK induced by NTHi**. **A**. A549 cells were inoculated with 10 and 50 MOI of NTHi, and phosphorayltion of p38 MAPK was detected by Immunoblot analysis. **B: **A549 cells were inoculated with 50 MOI of NTHi with or without 20 μM of SB203580, and phosphorayltion of p38 MAPK was detected by Immunoblot analysis. **A & B: **representative blots from three independent experiments.

To investigate the involvement of p38 MAPK signaling pathway, which is known as important component of TLR2-induced signaling pathway, NTHi-induced phosphorylation of p38 MAPK was, first, measured by using western blot analysis. As shown in Fig. 4A, the phosphorylation of p38 MAPK was induced within 15–30 min in A549 cells after NTHi inoculation, and the pre-treatment of the specific inhibitor of p38 MAPK SB203580 blocked p38 MAPK signaling activated by NTHi (Fig. 4B).

### Up-regulation of NF-kappa B DNA binding activity after NTHi stimulation

**Figure 5:**
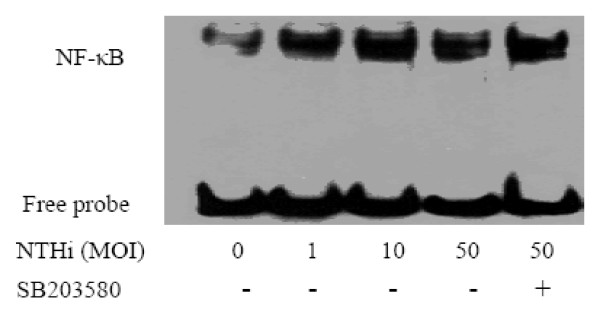
**Enhanced DNA binding activity of NF-kappa B induced by NTHi**. A549 cells were incubated with different concentrations of NTHi with or without SB203580, and DNA binding activity of NF-kappa B was measured by EMSA analysis. A representative gel from three independent experiments with similar results is shown in figure.

Since NF-kappa B signaling pathway is known as a component of TLR2 signaling pathway and down-stream of p38 signaling pathway, we investigated whether NTHi induces NF-kappa B activation in our system, and p38 MAPK is up-stream molecule of NTHi-induced NF-kappa B signaling pathway. EMSA analysis showed that NF-kappa B translocation and its DNA binding activity were markedly increased by NTHi within 90 mins, and DNA binding activity of NF-kappa B was not affected by pre-treatment of specific p38 MAPK inhibitor SB203580. These data indicate that p38 MAPK and NF-kappa B are two independent signal pathways in the regulation of host response of human lung epithelium against NTHi (Fig. 5).

### NTHi-induced COX-2 and PGE2 expression mediated by p38 MAPK and NF-kappa B signaling pathway

**Figure 6:**
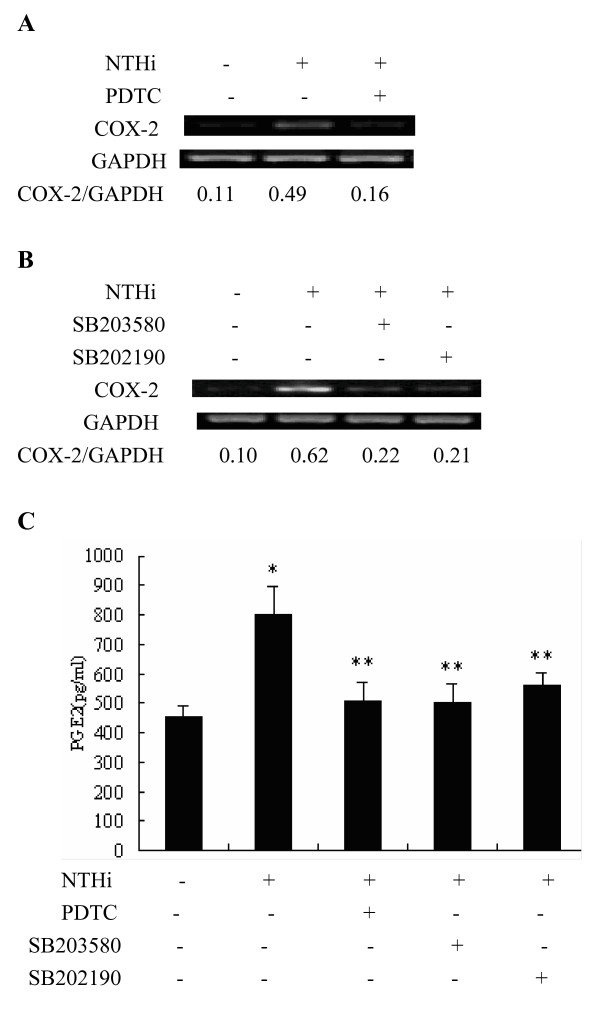
**NTHi-induced COX-2 mRNA and PGE2 expression mediated by p38 MAPK- and NF-kappa B-dependent signaling pathway**. **A: **A549 cells were inoculated with NTHi with or without PDTC, and COX-2 mRNA expression was measured by RT-PCR analysis 4 h after NTHi inoculation. **B: **A549 cells were inoculated with p38 inhibitors SB203580 or SB202190, and COX-2 mRNA expression was measured by RT-PCR analysis 4 h after NTHi inoculation. **C: **A549 cells were inoculated with NTHi with or without PDTC, SB203580, and SB202190, and expression levels of PGE2 were measured by ELISA assay 16 h after NTHi inoculation. Data are means ± SEM (n = 3). *p < 0.05 *vs*. Mock; **p < 0.05 *vs*. 50 MOI of NTHi.

To determine the role of NTHi-induced p38 MAPK and NF-kappa B activations in NTHi-induced COX-2 and PGE2 expression, the effects of p38 MAPK inhibitors SB203580 and SB202190 and NF-kappa B inhibitor PDTC on the NTHi-induced COX-2 mRNA expression and PGE2 production were measured by using RT-PCR analysis and ELISA assay, respectively. As shown in Fig. 6A &6B, NTHi-induced COX-2 mRNA expression was markedly inhibited by pre-treatment of NF-kappa B inhibitor PDTC and p38 MAPK inhibitors SB203580 and SB202190. Moreover, ELISA assay against PGE2 showed that NTHi-induced PGE2 expression was significantly inhibited by NF-kappa B inhibitor PDTC and p38 MAPK inhibitors SB203580 and SB202190. Taken together, these data clearly showed that NTHi-induced PGE2 expression is mediated by p38 MAPK- and NF-kappa B-dependent signaling pathway (Fig. 6C).

## Discussion

Although NTHi is an important respiratory pathogen in both children and adults, little is known about its molecular interaction with human lung epithelial cells. Equipped with transmembranous and cytosolic pattern recognition receptors, respiratory epithelium actively participates in immune reaction against invading pathogens instead of being only a passive physical barrier [10]. It has been shown that *Streptococcus pneumoniae*, *Chlamydia pneumoniae*, *Moraxella catarrhalis*, and respiratory syncytial virus induced COX-2 expression in pulmonary epithelium [9,11-13]. However, whether NTHi infections have effect on the expression of COX-2 and subsequent PGE2 in lung epithelial cells still remains uncertain.

Lipid products of arachidonic acid including prostaglandins play important roles in pulmonary immune regulation. PGE2 released by lung cells is demonstrated as a potent endogenous anti-inflammatory mediator, which modulates host immune response [7]. But, data about the molecular mechanisms of pulmonary PGE2 expression are very limited. In the present study, we demonstrated that NTHi induced COX-2 mRNA but not COX-1 mRNA in a dose-dependent manner in A549 cells. Furthermore, NTHi-induced PGE2 release was significantly suppressed by COX-2 specific inhibitor, but not COX-1 inhibitor. These results indicated that PGE2 release depends on COX-2 activity, in line with the previous studies by Schmeck *et al*. and Rupp *et al*. [9,14].

TLRs are well known cell surface receptors recognizing invading microbes and initiating cellular signaling pathways against bacterial components. Among of 11 mammalian TLRs, TLR2 is recognized as an important receptor for NTHi-induced signaling pathway in lung epithelial cells. Taken advantages of stably overexpressing TLR2 and TLR4 cells and TLR2 and TLR4 knock-out mice, we showed here that NTHi-induced COX-2 expression was mediated by TLR2 signaling pathway both *in vitro *and *in vivo*, but independently from TLR4. These data are in line with previous report that TLR2 is of particular important receptor for NTHi components [15,16].

The lung epithelial cell signaling networks activated by NTHi have been partially elucidated in recent years. Among these signaling pathways, p38 MAPK and NF-kappa B are two key molecules which coordinate the induction of multiple genes encoding inflammatory mediators and are involved in host immune responses to NTHi infections [2]. Activation of p38 MAPK up-regulates inflammatory cytokines, mucin MUC5AC and down-regulates TLR2 expression while activation of NF-kappa B increases the expression of IL-8, IL-1β, mucin MUC2 and TLR2 [15-19]. In addition, there exists an auto-regulation of NF-kappa B activation: NF-kappa B is essential for induction of cylindromatosis (CYLD) that in turn inhibits NF-kappa B signaling [20]. However, whether p38 and NF-kappa B are involved in COX-2 and PGE2 production induced by NTHi remained unclear. Herein, we showed that NTHi activated the phosphorylation of p38 MAPK and nuclear translocation of NF-kappa B within 90 mins, and the enhanced DNA binding activity was observed in nuclear extracts of A549 cells after NTHi inoculation. NTHi-induced COX-2 mRNA expression was markedly inhibited not only by p38 MAPK inhibitor SB203580, but also by another p38 MAPK inhibitor SB202190, and NF-kappa B inhibitor PDTC. Moreover, PGE2 release by NTHi, also, was significantly reduced by both p38 MAPK inhibitors and NF-kappa B inhibitor. The findings in the present study clearly demonstrate that the activation of p38 MAPK and NF-kappa B is involved in the regulation of COX-2 and PGE2 in NTHi-infected lung epithelium.

In this study, we found the impact of p38 MAPK on the activation of NF-kappa B. EMSA results showed that the NF-kappa B activation was not prevented by SB203580 in infected A549 cells, indicating that p38 MAPK and NF-kappa B are independent pathways regulating PGE2 expression in our experimental systems. Mancuso *et al*. reported that neither p38 nor ERK MAPK blockade had any effect on Group B streptococci (GBS)-induced NF-kappa B binding activity [21]. Vallejo *et al*. demonstrated that p38 inhibitor SB202190 inhibited GBS-induced activator protein (AP)-1-DNA activity, but did not prevent NF-kappa B activation [22]. Similarly, the study by Singer et al. showed that p38 MAPK and NF-kappa B may affect COX-2 expression in human airway myocytes via separate signaling pathways given the fact that SB203580 did not affect cytokine-stimulated IkappaBalpha degradation and NF-kappa B nuclear binding activity [23]. However, the above results cannot exclude another possibility that there exists an indirect cross-talk between p38 MAPK and NF-kappa B which converge further down in the signal cascades [21]. Indeed, there are still increasing evidences that the blockade of MAPK inhibited NF-kappa B-dependent gene transcription without affecting IkappaBalpha phosphorylation and NF-kappa B-DNA binding ability [9,24-27].

In our study, the fold induction of NTHi-induced PGE2 production was relatively low compared with those from other studies conducted by using *S. pneumoniae *or *C. pneumoniae *infection model [9,14], possibly due to the different signaling pathways mediated. However, the absolute amount of PGE2 production measured in our study in lung epithelial cells A549 is quite equivalent to those from other studies [28,29]. In addition to this, little is known about the patho-physiological role of the prostanoid intermediates including PGE2 in NTHi infections due to the absence of research information in this field. Thus, even though we could not rule the involvement of other prostanoid intermediates such as PGF and PGI in NTHi infections, we suggest here that NTHi-induced PGE2 may play an important role in NTHi infection. Further studies in the detailed molecular mechanisms underlying NTHi-induced PGE2 expression and in the pathological analysis of the role of PGE2 in NTHi infection based on the findings from present study will bring us novel insights into the therapeutic strategies of pulmonary NTHi infections.

## Conclusion

In conclusion, NTHi induces COX-2 and PGE2 expression in a p38 MAPK and NF-kappa B-dependent manner through TLR2 in lung epithelial cells *in vitro *and lung tissues *in vivo*. The full understanding of the role of endogenous anti-inflammatory PGE2 and its regulation will bring new insight to the resolution of inflammation in pulmonary NTHi infections.

## Competing interests

The author(s) declare that they have no competing interests.

## Authors' contributions

FX planned the experimental design, carried out Western blot, EMSA and drafted the manuscript. ZHX participated in cell culture and RT-PCR. RZ carried out NTHi isolation and identification. ZQW carried out ELISA. LJH performed *in vivo *mice experiments. KT carried out Q-PCR. JDL participated in the study design and helped to draft the manuscript. HHS participated in the study design and coordinated the research group. All authors read and approved the final manuscript.
